# Tiny Crustaceans, Big Story: Subfossil Cladocera and Multi-Proxy Sedimentary Records Reveal over 150 Years of Coupled Effects of Damming, Eutrophication and Aquaculture on a Yangtze Floodplain Lake in China

**DOI:** 10.3390/ani16162556

**Published:** 2026-08-16

**Authors:** Yingyi Cui, Rulin Lu, Cheng Peng, Huixin Ye, Yuling Xiao, Yan Li, Hanfei Yang, Giri Raj Kattel

**Affiliations:** 1 School of Geography and Remote Sensing, Guangzhou University, Guangzhou 510006, Chinamrllyaimfjq@163.com (R.L.); 19509689447@163.com (Y.X.); 2 Department of Infrastructure Engineering, The University of Melbourne, Parkville, Melbourne 3010, Australia; giri.kattel@unimelb.edu.au; 3 Department of Hydraulic Engineering, Tsinghua University, Beijing 100084, China

**Keywords:** Lake Zhangdu, cladoceran subfossil, community succession, environmental evolution, river–lake connectivity

## Abstract

Floodplain lake ecosystems around the world have witnessed growing environmental degradation under combined natural climatic variability and anthropogenic activities. Reconstructing their long-term ecological change is vital for lake ecosystem conservation and management. Cladoceran subfossils are used as an important proxy indicator to reconstruct ecological evolution of floodplain lake systems worldwide. The sedimentary record of Zhangdu Lake, a shallow floodplain lake in the middle and lower reaches of the Yangtze River in China, is important for reconstructing more than 150 years of environmental history and tracing the lake ecosystem’s transition from a natural flood-pulse system to a new regime driven by anthropogenic disturbances. Integration of subfossil cladoceran data with other biological proxies (diatoms and testate amoebae) and physicochemical sedimentary proxies successfully identified the period of river–lake disconnection and the associated ecological processes and succession in Lake Zhangdu. An abrupt disconnection of the lake from the Yangtze River in the early 1950s led to a sudden ecological vulnerability together with a drastic community decline. Sequential *t*-test Analysis of Regime Shifts (STARS) identified cladoceran community shifts in 1966 and 1999, with regime shift index (RSI) values of 1.195 and 3.29, respectively, reflecting multi-phase ecological reorganization following the 1950s disconnection. Differences among the proxies in the timing and magnitude of change and in their apparent drivers reflected group-specific habitat preferences, response thresholds, and trophic positions. This study shows that the assessment of multi-taxa paleoecological indicators can provide a strong evidence base for guiding restoration efforts in degraded floodplain lake ecosystems in China and elsewhere.

## Simple Summary

Many shallow lakes worldwide are exposed to various environmental problems, including water quality degradation and biodiversity losses due to increased human disturbance. Information regarding their long-term evolution is crucial for ecological protection. Using subfossil cladocerans preserved in the sediments of Lake Zhangdu in Wuhan, China, this study reconstructed the lake’s environmental history over the past 150 years, spanning natural connectivity with the Yangtze River, disconnection following dam construction, and subsequent seasonal water replenishment. Identifying the timing of ecological changes and the sensitivity of the Lake Zhangdu ecosystem to changes in river–lake connectivity can inform the restoration of shallow lake systems in China and elsewhere.

## 1. Introduction

The middle and lower reaches of the Yangtze River in China host multiple priority biodiversity conservation hotspots of global significance, designated by the World Wide Fund for Nature (WWF). The region also contains more than 600 shallow lakes and wetlands larger than 1 km^2^, accounting for more than 60% of the national total; some are Ramsar Wetlands of International Importance and are protected under different legislation [1]. Despite their protection, these wetlands are exposed to subtropical climate change, with frequent flood inundation as well as intensive anthropogenic disturbances. Since the 1950s, anthropogenic disturbances have intensified these wetlands, causing a range of environmental issues, including eutrophication, biodiversity losses and abrupt flood episodes [2]. Many naturally connected wetlands with the Yangtze River showing periodic flood-pulse events in the past have transformed into a new ecological regime. However, biological communities under natural background conditions are not well understood when they are exposed to impacts [3,4]. Impacts such as the large-scale sluice construction across the basin since the 1950s have disconnected over 99% of lakes in this region from the Yangtze River, leading to continuous nutrient accumulation in the lake system. Extended pollutant residence time within the lake system has impaired the water purification capacity [5,6], thereby exacerbating lake eutrophication [7]. Flooding, eutrophication, pollution and other anthropogenic impacts have led to sediment disturbance and the scarcity of historical records, including the preservation of remains of biological communities in lake sediments [8,9].

Multi-proxy analyses of chemical and biological remains in lake sediments (e.g., diatoms, cladocerans and geochemical elements) can effectively offset the shortage of long-term continuous lacustrine datasets of contemporary ecosystem monitoring and help reconstruct environmental changes, including ecological succession. Cladocerans, which belong to the class Branchiopoda, play an important role in maintaining water quality and structuring aquatic food webs at the middle of the trophic system [10,11]. Diatoms and testate amoebae constitute key components at the base of the lake food web, showing strong sensitivity to changes in water nutrient levels and hydrodynamic conditions, including water-level fluctuations [12,13]. Due to the presence of a chitinous and siliceous exoskeleton, these biological proxies have been widely used in palaeoenvironmental reconstructions, including the ecological evolution of floodplain lakes worldwide [14,15]. The use of a multi-proxy approach in lakes is now validated to provide a scientific basis for the management and restoration of lake ecosystems worldwide [16,17,18,19].

Lake Zhangdu, part of the Yangtze River floodplain system, has a history of natural connectivity with the river, supporting significant ecosystem services such as fisheries and wildlife [20]. However, numerous dams and sluices have been constructed since the 1950s, resulting in a reduction in the lake area and alterations to its hydrological regime. A multi-proxy analysis of diatoms, elemental geochemistry and grain size in this lake over the past 150 years is fundamentally crucial, as it would serve as an extension of the previous studies by Qin et al. on testate amoebae [21] and Kattel et al. [22] on subfossil cladocerans to validate the community succession, ecosystem shift and ecological evolution. Building on the 1863–2011 cladoceran record previously reported by Kattel et al. [22], this study adds new cladoceran data for 2013–2018 from a second sediment core collected in 2018. These new data allow us to examine cladoceran community changes during the period following the seasonal river–lake connectivity restoration initiated in 2005. This study further integrates cladoceran data with the results from sedimentary physicochemical properties and fishery records to explore cladoceran response to lake eutrophication, dam construction and fishery development in the area. The study compares the responses of subfossil cladocerans with the responses of diatoms and testate amoebae to validate the occurrences of ecosystem shift over the past 150 years. This study contributes to the evidence-based conservation, management and resource utilization of China’s Yangtze River floodplain lake system, including Lake Zhangdu.

## 2. Materials and Methods

### 2.1. Overview of the Study Area

**Figure 1:**
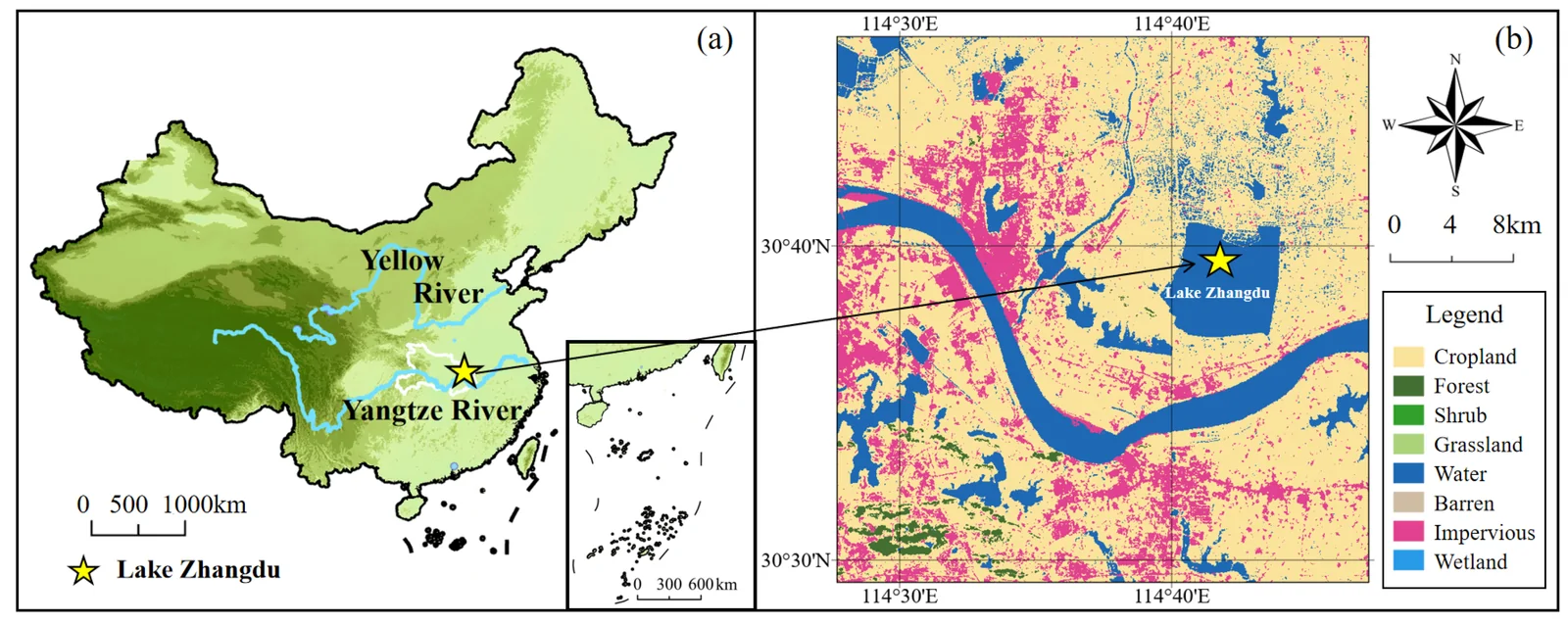
Map of Lake Zhangdu and its surroundings: (**a**) location of Lake Zhangdu in China; (**b**) land use surrounding Lake Zhangdu. The land-use dataset was derived from the 30 m annual China Land Cover Dataset (CLCD) [23].

Lake Zhangdu is in the middle reaches of the Yangtze River, approximately 1 km from the river, and covers an area of 35.2 km^2^. It lies within a subtropical monsoon climate zone, with an average annual precipitation of 1200 mm and an average annual temperature of 16.3 °C. In 2023, the land types surrounding Lake Zhangdu included cropland, forest, shrubland, grassland, water bodies, bare land, impervious surfaces, and wetlands (Figure 1). The land-cover classification data were derived from the 30 m annual China Land Cover Dataset (CLCD) developed by Jie Yang and Xin Huang [23]. The cropland category in this study strictly follows the unified classification standard of the CLCD dataset, which defines cropland as artificial land primarily used for crop cultivation. Current assessments of the lake’s Total Nutrient Load Index (TLI) indicate mild eutrophication, characterized by severe pollution in the southwestern region and exacerbated pollution during the dry season in terms of both time and space [24].

Historically, Lake Zhangdu has long been connected to the Yangtze River. The lake’s surface area and water level have fluctuated significantly with the seasons, making it an important flood retention area for the Yangtze River [22]. By the 1950s, human development activities in the lake area intensified. In addition to increased fishing pressure, large-scale land reclamation and water conservancy projects were carried out in the Lake Zhangdu area, resulting in the severing of the connection between the lake and the river. Consequently, the lake’s area rapidly shrank, and its water level dropped and stabilized. The flux of material exchange between the lake and the river decreased, pollution caused by human activities accumulated, eutrophication became an increasingly serious problem, the movement of organisms was obstructed, and the number of fish species decreased by 42.5% compared to before the separation [25,26]. In 2002, Lake Zhangdu was designated a wetland nature reserve by the WWF, and the local government launched a series of wetland conservation initiatives in the area, primarily aimed at restoring the Yangtze River’s web of life. In 2005, seasonal operation of the sluice gate was initiated to divert Yangtze River water and wild fish fry into Lake Zhangdu, thereby partially restoring hydrological connectivity between the lake and the river. In 2007, it was selected as one of the first batches of wetland nature reserves in the middle and lower reaches of the Yangtze River. As the wetland habitat continues to improve, it has attracted tens of thousands of waterbirds to nest, stop over, or overwinter there [27].

### 2.2. Sample Collection and Laboratory Analysis

In April 2011, our research team collected a 45 cm sediment core from the center of Lake Zhangdu (30°39′ N, 114°42′ E) using a gravity sampler. The chronology of this core, spanning 1863–2011, together with its diatom, geochemical, and grain-size records, was reported by Zhang et al. [20]. The cladoceran record from the same core was previously reported by Kattel et al. [22].

In 2018, our research team collected a second, 50 cm sediment core from the same location. This core was not independently dated using radionuclides. The collection year of 2018 was used as the nominal age of the top of the recovered core. The uppermost 5 cm was provisionally regarded as representing approximately 2013–2018 based on the recent mean sediment accumulation rate of about 1 cm year^−1^ derived from the independently dated 2011 core. This rate was used only as a first-order estimate and was not considered evidence of constant linear sediment accumulation. Comparisons of the loss-on-ignition and grain-size profiles between the two cores were used only to assess broad stratigraphic consistency and did not provide independent chronological control. Possible non-linear sediment accumulation, top-core compaction, and disturbance of the sediment–water interface during gravity coring could not be quantified. The assigned ages are therefore approximate, and the 2018 core samples are treated only as a tentative extension of the recent cladoceran record.

The new 2013–2018 samples were processed and analyzed by our research team at Guangzhou University using the procedures described below. Cladoceran analyses for sample pretreatment, microscopic identification, and counting followed the procedure described by Frey [28]. In the laboratory, approximately 5 g (wet weight) of thoroughly mixed sample was weighed for analysis, following the procedure described by Frey in 1986 [28]. First, the sample was placed in a beaker containing 200 mL of a 10% KOH solution; then, the sample was heated in a 60 °C water bath for 1 h to break up flocs. The sample was then filtered through an 80 μm sieve; the material retained on the sieve was washed with distilled water and collected, and the volume was finally adjusted to 10 mL with the addition of a small amount of formaldehyde solution to inhibit decomposition. To aid in identification, the chitin residues were stained with a carmine–glycerol solution.

The identification of cladocerans was conducted under an optical microscope. Before beginning, the sample was thoroughly shaken; then, 100 μL of the suspension was transferred into a counting chamber, and identification and counting were performed under a microscope at magnifications ranging from 100× to 400×. The identification criteria for cladocerans were based on current authoritative international and domestic literature [29,30]. Given that cladoceran microfossils typically appear only as partial body structures, the count for a specific species or genus must be conducted separately for each type of fragment, with the most abundant fragment type ultimately serving as the basis for the species or genus count. The total number of individuals counted per sample must be at least 100. In addition, the abundance of cladocerans was converted into relative abundance (i.e., the proportion of a specific cladoceran species in the total number of cladocerans). Percentage changes in cladoceran communities were plotted using Tilia v3.0.2 software, and were divided in zones based on CONISS cluster analysis [31].

The diatom dataset used for the multi-proxy comparison was generated previously by members of our research group from the 2011 sediment core and was reported by Zhang et al. [20]. In that study, approximately 0.5 g of sediment was treated with 10% HCl to remove carbonates and digested with H_2_O_2_ to eliminate organic matter. After centrifugation and repeated rinsing with distilled water, the residue was mounted on slides. Diatom identification followed Krammer and Lange-Bertalot [32]. The complete taxonomic composition and relative-abundance data are available in Zhang et al. [20].

**Figure A1:**
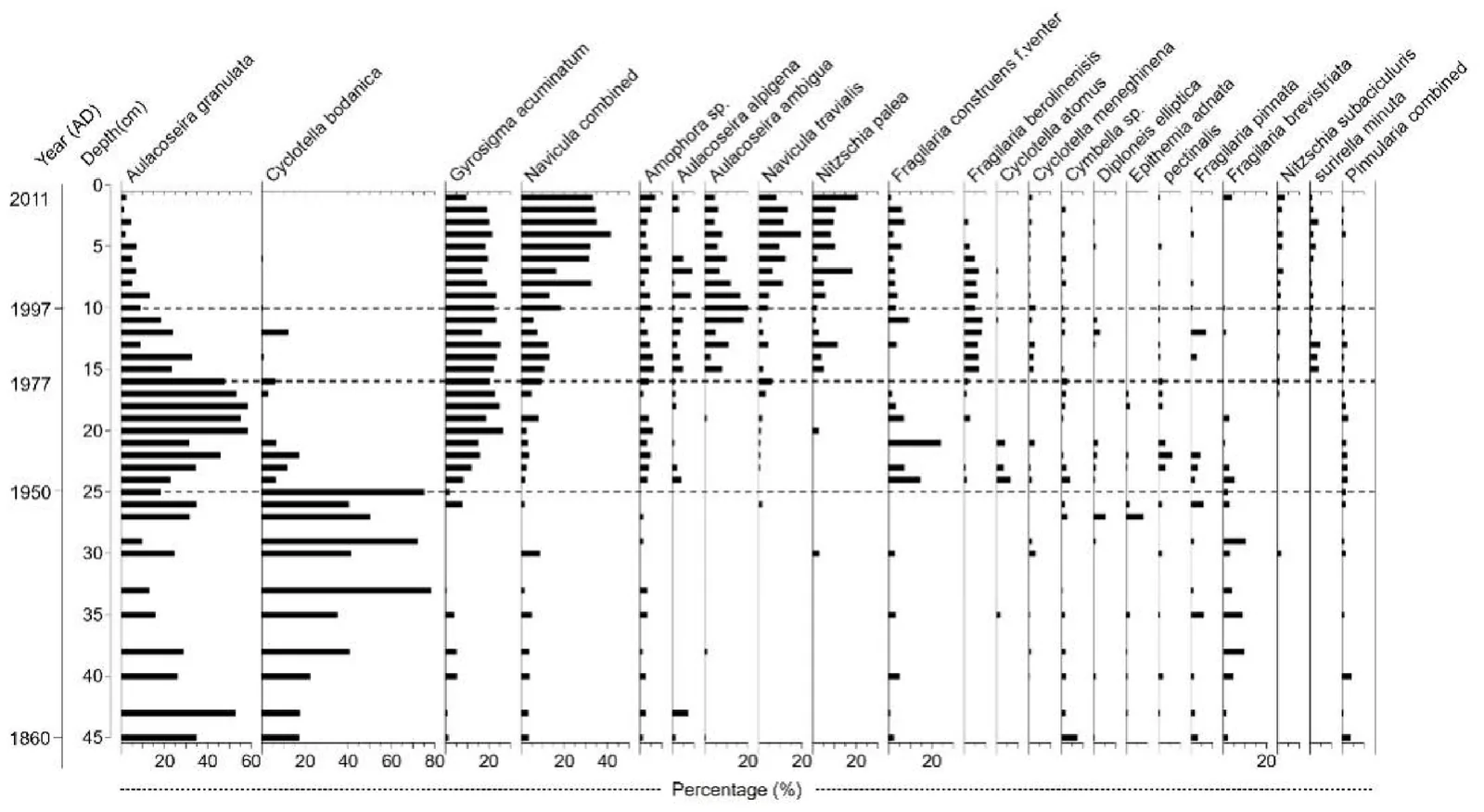
Temporal trend of the diatom community in the sedimentary core of Lake Zhangdu from 1860 to 2011. Data source: Zhang et al. [20]. The dotted lines in the figure represent the boundary lines demarcating the different zones based on the succession phases of the cladoceran community.

**Figure A2:**
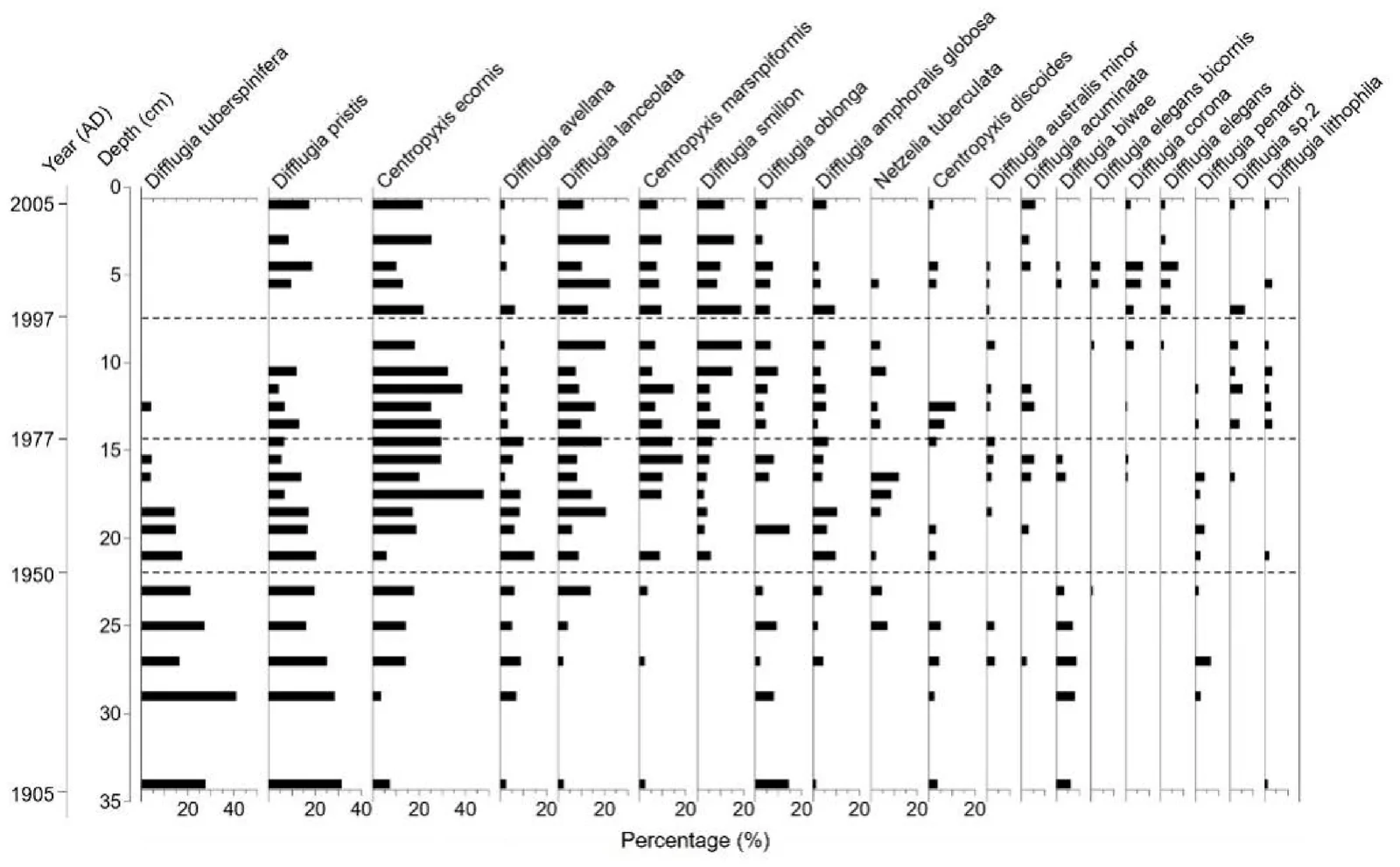
Temporal trend of the testate amoeba community in the sedimentary core of Lake Zhangdu from 1905 to 2005. Data source: Qin et al. [21]. The dotted lines in the figure represent the boundary lines demarcating the different zones based on the succession phases of the cladoceran community.

The testate amoeba data were obtained from Qin et al. [21]. In that study, testate amoebae were extracted from sediment subsamples following Scott and Medioli [33]. The material was rinsed with distilled water and wet-sieved through 500 μm and 35 μm sieves before microscopic identification. The temporal distributions of diatoms and testate amoebae are presented in Appendix A, Figure A1 and Figure A2, respectively.

Sedimentary geochemical and grain-size data (median diameter, MD) were obtained from Zhang et al. [20], whereas fish-yield data were obtained from Xu et al. [34].

### 2.3. Data Analysis

Detrended Correspondence Analysis (DCA) can effectively eliminate the bow-shaped effect in ordination and reveal trends in biological community changes [35]. In this study, after Hellinger normalization of the relative abundance data for genera and species across the three biological groups, DCA analysis was performed using CANOCO 5.0 software [36]. Scores along the first axis (DCA1) were extracted as the primary indicator of long-term changes in community structure across each group and were used for subsequent detection of community shifts and correlation analysis with environmental factors. At the same time, based on the gradient lengths output by DCA, statistical grounds were provided for model selection in the subsequent Redundancy Analysis (RDA). Based on the DCA1 score sequences, Spearman’s rank correlation analysis was used to examine the associations between community structure and environmental factors, with *p* < 0.05 indicating a significant correlation and *p* < 0.01 indicating a highly significant correlation. Prior to correlation analysis, Shapiro–Wilk normality tests were conducted on all variable sequences. The test results showed that most environmental variables did not conform to the normal distribution required for parametric correlation; hence, the non-parametric Spearman’s rank correlation was adopted instead of Pearson correlation to ensure statistical robustness. For fish-yield data, missing values were imputed using the minimum value for calculations.

Sequential *t*-test Analysis of Regime Shifts (STARS) can test for significant differences in the mean of a time series point by point using a sliding window, objectively identifying nodes of community regime shifts and quantifying the intensity of these shifts; it is an important method for analyzing regime shifts in paleoecological time series [37]. This study utilized the Excel VBA macro add-in for time-series steady-state shift detection developed by Rodionov to perform all STARS computational analyses [38]. Using the DCA1 score time series of three types of biological communities as input data and combining the temporal resolution of sediment cores from the study area with standard settings for paleoecological studies of lakes in the middle and lower reaches of the Yangtze River, the following core parameters were determined: sliding window length *l* = 10, significance level α = 0.05, and a test for homogeneity of variances based on the standard deviation of the l samples preceding the window. Additionally, the input time series underwent pre-whitening to eliminate the interference caused by autocorrelation. The analysis using the add-in ultimately yielded two key results: (1) the years of significant shifts in the DCA1 time series for the three types of biological communities, identifying the key time points for community regime shifts; and (2) the Regime Shift Index (RSI), which quantifies the cumulative degree to which observed values deviate from the mean of the current regime and serves as a threshold criterion for determining significant community shifts.

This study selected the cladoceran DCA1, along with key heavy metal indicators, such as Mg, Fe, Zn, Mn, Pb, and Cu, and combined them with total organic carbon (TOC), total phosphorus (TP), frequency-dependent magnetic susceptibility (χfd), loss on ignition, and grain size. Spearman’s rank correlation analysis was used to preliminarily investigate the relationships among these indicators. Redundancy Analysis (RDA) is primarily used to investigate linear relationships between multivariate response data and explanatory variables, and to identify key environmental variables influencing changes in the cladoceran community [39]. For the redundancy analysis, cladoceran genera and species that occurred in at least two samples and had a concentration greater than 1% were selected as response variables, while environmental factors identified through correlation analysis served as explanatory variables. Significant factors explaining cladoceran community succession were progressively preselected based on a Monte Carlo permutation test (*p* < 0.01; *n* = 999). The aforementioned numerical analyses were also performed using CANOCO 5.0 software.

The Shannon–Wiener index was used to characterize the diversity of the cladoceran community, and the Sørensen dissimilarity index (βsor) was employed to reflect the overall differences in cladoceran species composition among samples taken at different times (the diversity indexes above were calculated following [40]). The planktonic-to-littoral cladoceran ratio (P/L) was calculated as the summed relative abundance of planktonic taxa divided by the summed relative abundance of littoral taxa. It can indicate changes in lake water level and associated shifts in cladoceran habitat structure [41].

## 3. Results

### 3.1. Cladocera Community Composition and Succession Process

**Figure 2:**
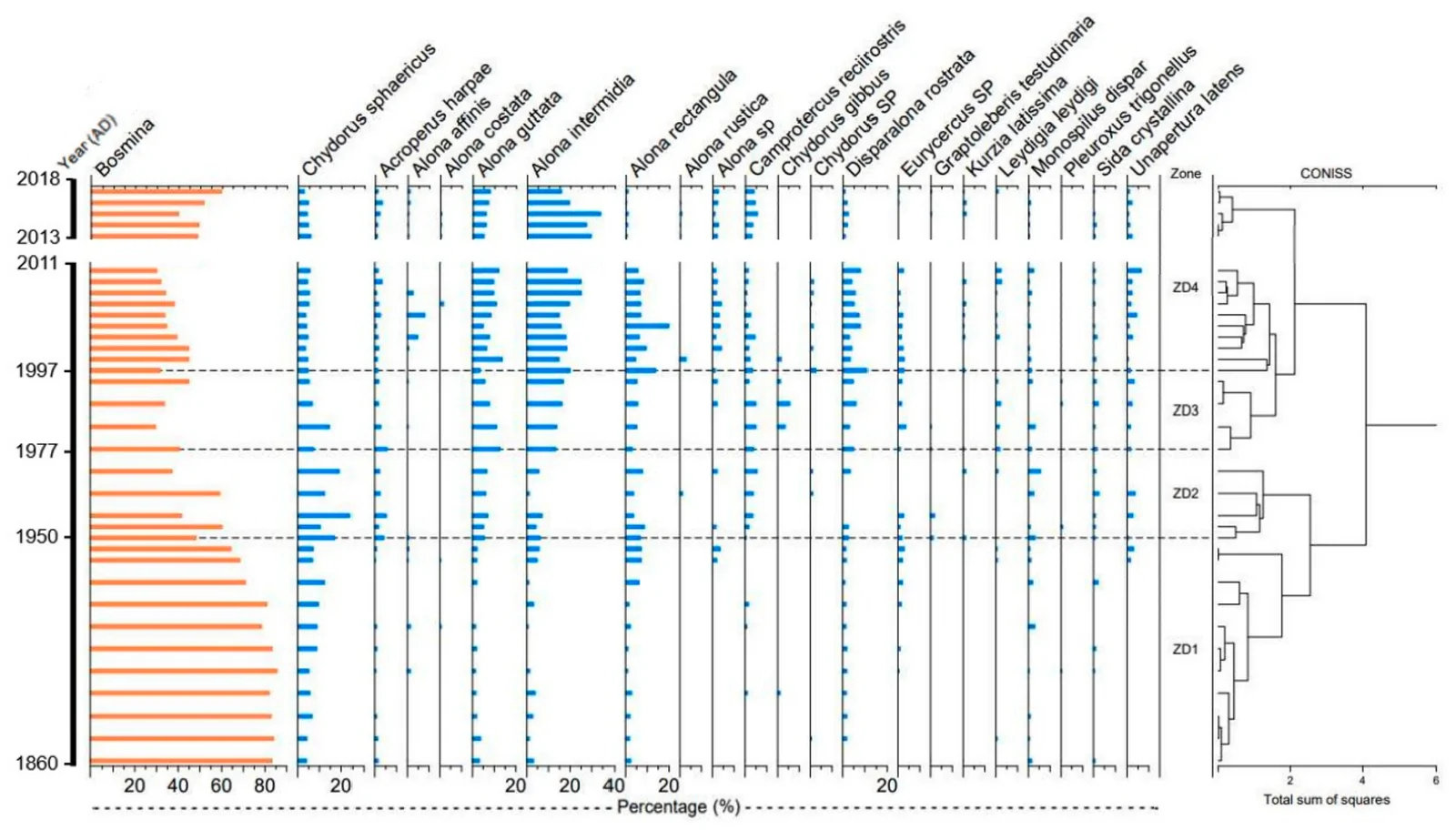
Temporal changes in the cladoceran community of Lake Zhangdu over the past 150 years. The 1863–2011 data were previously reported by Kattel et al. [22], whereas the 2013–2018 data were newly generated in this study from the 2018 sediment core. The 2013–2018 interval was treated as a continuation of ZD4, during which the community structure remained broadly stable.

A total of 36 cladoceran taxa were identified in the sediment core from Lake Zhangdu (Figure 2). Among them, *Bosmina* was the dominant group, with an average relative abundance of 54%, occupying a dominant position in the lake ecosystem. *Alona* was the subdominant group, mainly including *Alona intermedia*, *Alona rectangula*, and *Alona guttata. Chydorus sphaericus* was a common species, with an average abundance of approximately 8%. CONISS analysis indicated that cladoceran community succession over the past 150 years could be divided into four phases (Figure 2).

ZD1 (1863–1950): During this phase, the planktonic *Bosmina* was highly dominant, with a maximum relative abundance of 85.1%. *C. sphaericus* was the subdominant species, showing an increasing trend and reaching a peak of 16.9%. The abundances of littoral and benthic species were generally low. The mean P/L ratio was as high as 8.47, indicating that the lake was in a high-water-level and strong hydrodynamic environment.

ZD2 (1950–1977): The community was still dominated by *Bosmina* and *C. sphaericus*, but the abundance of *Bosmina* decreased substantially, with a peak of only 55.8%, while *C. sphaericus* increased to 23.1%. The proportion of littoral species increased significantly, and the dominance of planktonic species began to weaken. The P/L ratio dropped sharply to 1.78, reflecting a notable decline in water level and a marked weakening of hydrodynamic conditions.

ZD3 (1977–1997): *A. intermedia* replaced *C. sphaericus* as the subdominant species. The average abundance of *Bosmina* further decreased to 33%, while the proportions of littoral and benthic species continued to increase. *C. sphaericus* showed an initial increase followed by a decrease. The P/L ratio continued to decline to 0.83, and the water level and hydrodynamic conditions remained at relatively low levels.

ZD4 (1997–2018): The community was centered on *Bosmina* and *A. intermedia.* The abundance of *Bosmina* stabilized at 33%, while *A. intermedia* increased to 20%. Non-planktonic species, including *A. guttata, A*. *rectangula*, and *Disparalona rostrata*, became highly dominant. The P/L ratio remained very low, with a mean value of 0.81, indicating the continued dominance of littoral and benthic habitats. The period 2013–2018 showed a broadly similar community composition and was treated as an approximate recent extension of ZD4. Within these uppermost samples, the community structure remained generally stable, while the relative abundance of *Bosmina* increased toward the core surface.

### 3.2. Variation Trends of Multiple Sedimentary Indicators and Historical Fish Catches

**Figure 3:**
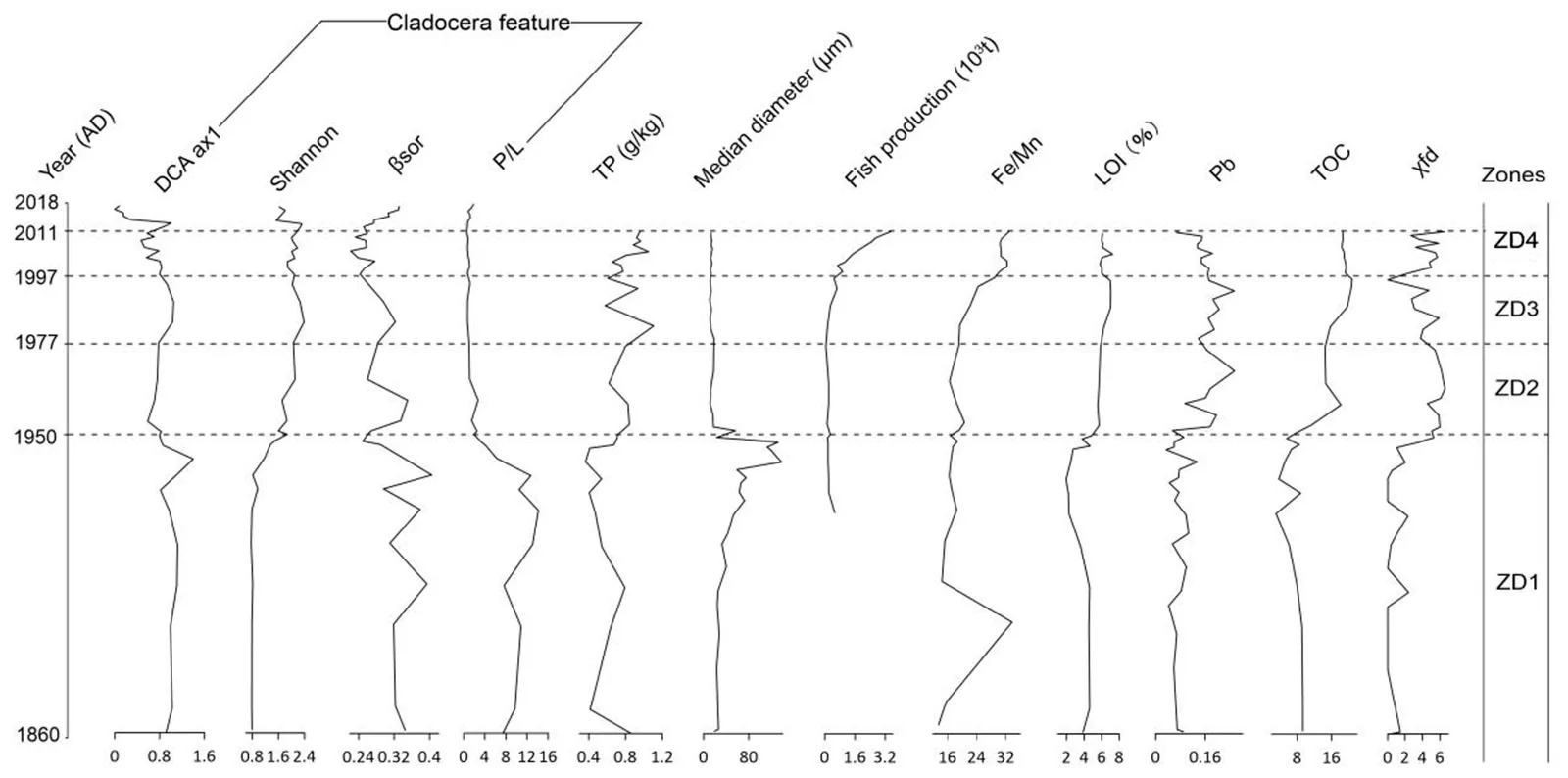
Cladoceran community features (DCA1, Shannon index, total β-diversity, P/L), sedimentary environment indicators, and fishery yield indicators (fish production). Dotted lines delineate the zones based on cladoceran succession. Sedimentary proxy data were obtained from Zhang et al. [20], and fish-yield data were obtained from Xu et al. [34]. Fish-yield data are available only up to 2011.

As shown in Figure 3, the characteristic indicators of cladocera, including DCA axis 1 scores, diversity parameters, and multiple sedimentary parameters, showed enough consistency that they could be divided into four corresponding phases (Figure 3).

ZD1 (1863–1950): During this phase, the DCA1 score remained stable at a low level of 0–0.4 over a long period, with extremely small fluctuations. The Shannon–Wiener index remained at a low level over the long term, with only a slight increase around 1940. The βsor index fluctuated frequently and substantially within the range of 0–0.4. TP remained stable at a low level of 0.4–0.8 g/kg, with no significant accumulation. The P/L ratio fluctuated greatly between 4 and 16, remaining at a high level overall. The MD of sediments remained at a high level, reaching a maximum of 137 μm, fluctuating significantly with hydrological changes. Fish yield remained below 0.5 × 10^3^ t over the long term without an obvious increasing trend. The Fe/Mn ratio exhibited considerable fluctuations but remained at a relatively high level overall. Loss on ignition (LOI), Pb, TOC, and χfd all exhibited decreasing trends, although both Pb and χfd displayed considerable fluctuations.

ZD2 (1950–1977): The DCA1 score of cladocera showed a significant jump in 1950, rising continuously from 0.4 to about 0.8. The Shannon–Wiener index increased rapidly from 1.2 to about 2.0. The fluctuation amplitude of the βsor index narrowed significantly, gradually decreasing from 0.4 to about 0.2. TP showed a continuous and rapidly increasing trend, rising from 0.8 g/kg to 1.2 g/kg. The P/L ratio showed a precipitous decline, dropping sharply from above 8 to below 2 and continuing to decrease. The MD decreased rapidly from 56 μm to below 20 μm. Fish yield rose slowly from 0.5 × 10^3^ t to 1.6 × 10^3^ t, showing a steady increasing trend. The Fe/Mn ratio, LOI, Pb, TOC, and χfd began to increase, while Pb consistently showed marked fluctuations.

ZD3 (1977–1997): The DCA1 score continued its rising trend, from 0.8 to about 1.0, with a slight deceleration in the growth rate. The Shannon–Wiener index first rose to a peak of 2.4 and then declined rapidly, generally remaining in the range of 1.6–2.0. The βsor index showed a slight increase in fluctuation amplitude, but overall continued its declining trend, remaining between 0.1 and 0.3. TP fluctuated substantially at a high level of 1.0–1.2 g/kg, with an overall pattern of first increasing and then decreasing. The P/L ratio stabilized at a very low level of about 0.8, with no significant fluctuations. The MD remained at a low level below 20 μm. Fish yield showed explosive growth, rising rapidly from 1.6 × 10^3^ t to 3.0 × 10^3^ t. The trends of the Fe/Mn ratio, LOI, Pb, and TOC continued their previous patterns, whereas χfd decreased slightly.

ZD4 (1997–2018): The DCA1 score remained at a high level of 1.0–1.2, stabilizing after minor fluctuations. The Shannon–Wiener index stabilized at a moderate-to-high level of 1.8–2.0. The βsor index stabilized at a low level of 0.1–0.2, with no large fluctuations. TP again showed a significant increasing trend, with a peak approaching 1.2 g/kg, reaching the highest level in nearly 150 years. The P/L ratio remained at a low level of about 0.8, with no obvious recovery. The MD remained at a low level of about 15 μm, with only minor fluctuations. Fish yield rose to above 3.2 × 10^3^ t, reaching a historical peak. The Fe/Mn ratio continued to rise, LOI and TOC remained stable, Pb exhibited a clear decreasing trend, and χfd showed a fluctuating increase. Across the uppermost 5 cm of the 2018 core, provisionally corresponding to 2013–2018, DCA1 scores and the Shannon–Wiener index decreased toward the core surface, with DCA1 scores falling below 0.2. The βsor index increased, whereas the P/L ratio remained low at approximately 0.8 and showed only a slight increase.

### 3.3. Relationships Between Cladocera Indicators and Other Eco-Environmental Parameters

**Figure 4:**
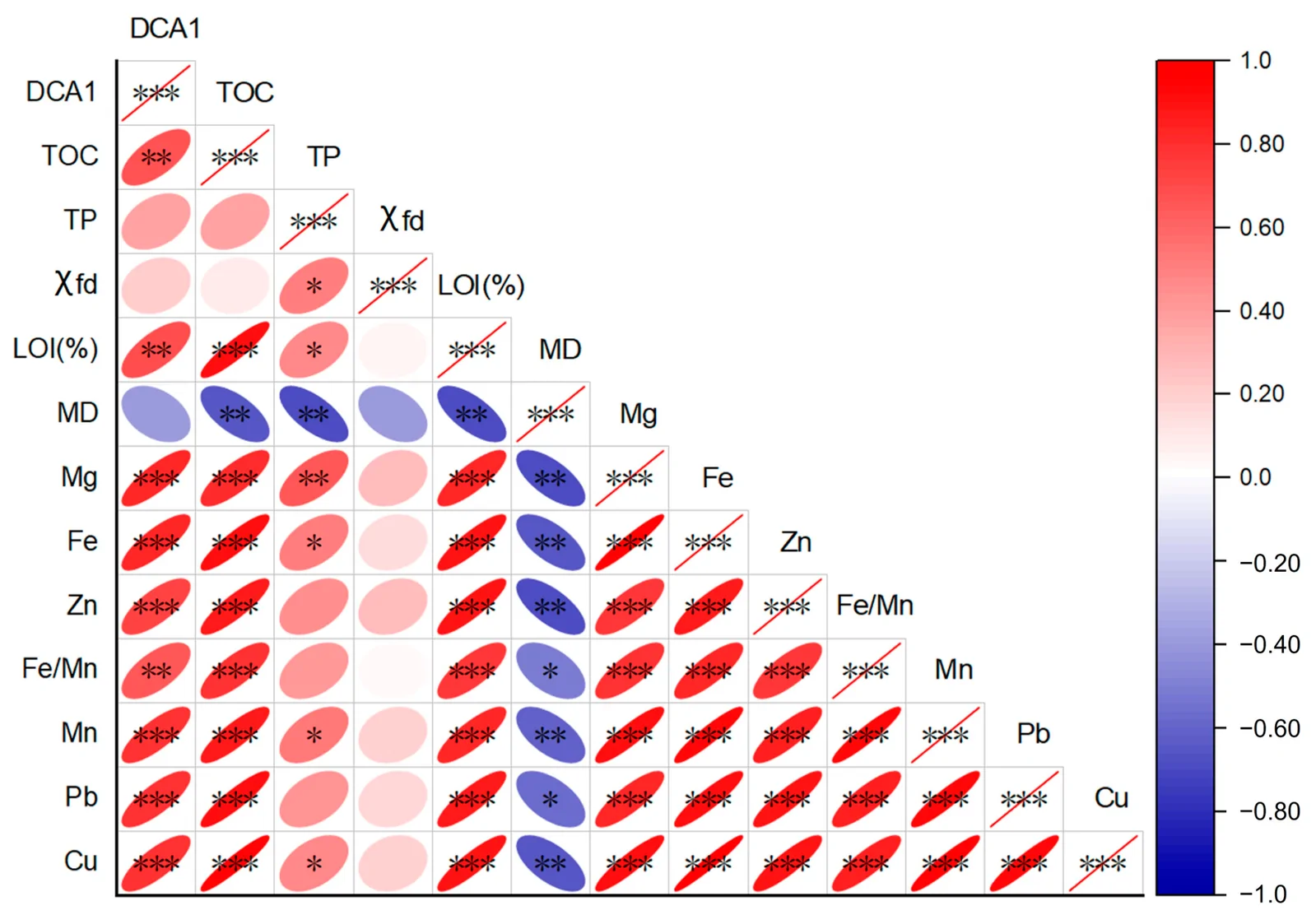
Spearman’s rank correlation between cladoceran DCA1 scores and sedimentary proxies. (Note: * *p* ≤ 0.05; ** *p* ≤ 0.01; *** *p* ≤ 0.001).

Significant correlations were observed between DCA1 scores and geochemical indicators in various regions. Specifically, DCA1 showed a highly significant positive correlation (*p* < 0.01) with Mg, Fe, Pb, Mn, Cu, and Zn, with correlation coefficients ranging from 0.74 to 0.83; as DCA1 values increased, the concentrations of these metallic elements increased in tandem. DCA1 also showed a highly significant positive correlation with LOI, TOC, and Fe/Mn, with correlation coefficients ranging from 0.65 to 0.70; however, the strength of these correlations was slightly weaker. DCA1 was positively correlated with TP, with a correlation coefficient of 0.37 (*p* < 0.05). DCA1 showed no significant association with χfd and only a weak, nonsignificant negative correlation with MD (Figure 4).

Extremely strong positive correlations were observed among the core elements associated with DCA1 (Mg, Fe, Zn, Pb, Cu, Mn, and Fe/Mn). The Spearman correlation coefficients between each pair exceeded 0.76, with *p*-values less than 0.01. Among these, the correlation coefficient between Fe and Cu reached 0.97, the correlation coefficient between Mg and Fe reached 0.96, and that between Mn and Fe/Mn reached 0.94, indicating that changes in the concentrations of these elements are highly synchronized. At the same time, Mg, Fe, Zn, Pb, Cu, Mn, and Fe/Mn also showed extremely strong positive correlations with TOC and LOI, with correlation coefficients all above 0.76 and *p*-values less than 0.01. For example, the correlation coefficient between Fe and TOC was 0.90, and the correlation coefficient between Zn and LOI was 0.89, indicating that changes in the concentrations of these elements are highly consistent with organic matter indicators.

MD showed strong negative correlations with TOC, LOI, TP, Mg, Fe, Zn, Mn, Pb, Cu, and Fe/Mn (*p* < 0.05); specifically, the correlation coefficients between MD and LOI and Zn were both −0.70. As the concentrations of TOC, LOI, Mg, Fe, and other indicators increased, MD generally showed a decreasing trend. There was no significant correlation between MD and χfd. TOC was extremely significantly positively correlated with LOI and various metal indicators, including Mg and Cu (*p* < 0.05); the correlation coefficient between TOC and Cu reached 0.95, indicating a high degree of synchrony between organic matter and metal element distribution. TP was significantly positively correlated with Mg, Fe, Mn, and Cu; χfd was significantly positively correlated only with TP, while showing weak correlations with the remaining indicators.

**Figure 5:**
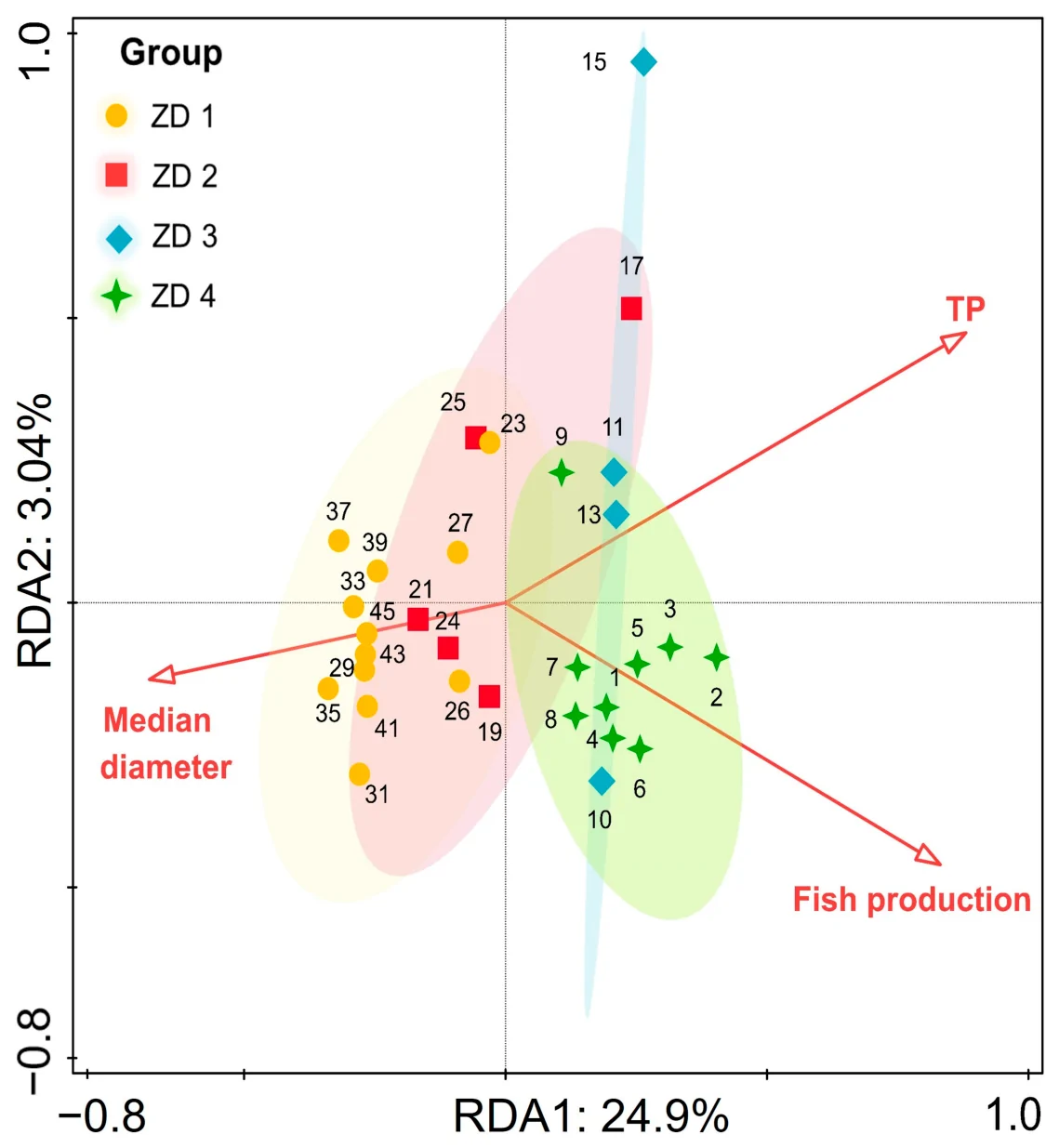
Redundancy analysis exploring the relationship between the cladoceran community and environmental parameters. The numbers in the figure represent sample depths (cm).

Redundancy analysis (RDA) revealed that sediment MD, TP, and fish yield were the most important factors influencing cladocera community variation. The first and second RDA axes (RDA1, RDA2) explained 24.9% and 3.04% of the total variation in the community structure, respectively, with a cumulative explanation of 27.94% (Figure 5). The primary variation in community structure was concentrated on the first ordination axis, and the communities of the four groups exhibited a gradient differentiation pattern along the RDA1 axis. Specifically, samples from group ZD1 were mainly distributed on the negative side of the RDA1 axis, showing a significant difference in community structure from groups ZD3 and ZD4. Group ZD2 was in an intermediate transition zone from near the origin to the weakly positive side of the RDA1 axis, representing a transitional phase in the community gradient differentiation. The arrows of TP and fish yield pointed in the same direction and showed a strong positive correlation, making them the core environmental drivers of the community structural characteristics of groups ZD3 and ZD4. The arrow lengths of TP and MD were longer, indicating that these two factors had a higher explanatory power for community structure than fish yield (Figure 5).

### 3.4. Response Patterns of Multiple Biological Communities to Environmental Change

**Figure 6:**
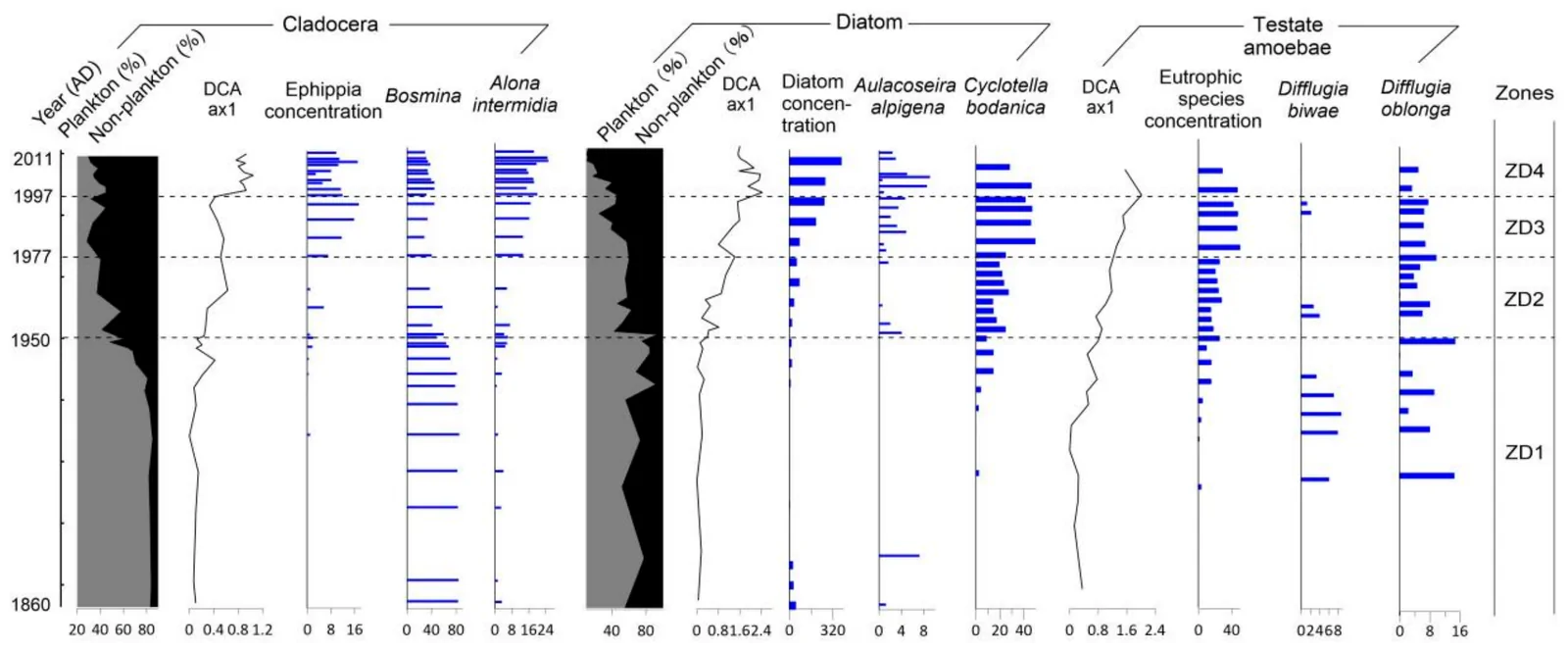
Distribution of the most abundant taxa in three biological groups in Lake Zhangdu.

The main genera and dormant eggs of cladocera, diatoms and testate amoebae are distributed as follows (Figure 6):

ZD1 (1863–1950): Both cladocera and diatoms were dominated by planktonic species, and the concentration of dormant eggs was low. The concentration of diatoms was low overall, the abundance of eutrophic indicator species *Aulacoseira alpigena* was low, and the abundance of plankton species *Cyclotella bodanica* was high. The content of eutrophic species in testate amoebae was extremely low; the abundance of *Difflugia biwae*, which indicates an oligotrophic environment, reached the highest value in history; and the abundance of *Difflugia oblonga*, which reflected the eutrophication process of lakes, decreased significantly.

ZD2 (1950–1977): The proportion of planktonic cladocera and diatoms decreased significantly, while non-plankton species dominated and the concentration of dormant eggs increased. The absolute concentration of diatoms increased slightly but remained at a low value, and the abundance of *C*. *bodanica* decreased significantly. The concentration of eutrophic testate amoebae increased, the abundance of *D. biwae* decreased significantly, and the abundance of *D. oblonga* peaked around 1950 and then decreased slightly before remaining relatively stable.

ZD3 (1977–1997): The proportion of plankton in cladocera remained basically stable, and the concentration of dormant eggs rose to a peak and then declined. The proportion of planktonic species of diatoms further decreased, the non-plankton species maintained dominance, the abundance of *A. alpigena* increased and reached its peak, and the abundance of *C. bodanica* remained at a low level. The concentration of eutrophic testate amoeba reached its peak, *D. biwae* almost disappeared, and *D. oblonga* remained stable.

ZD4 (1997–2018): Non-planktonic species of cladocera and diatoms had absolute dominance, and the concentration of dormant eggs was at a high level. The dormant eggs of diatoms rose sharply to a peak, the abundance of *A. alpigena* decreased, and *C. bodanica* disappeared. The concentration of eutrophic testate amoebae decreased rapidly, *D. biwae* disappeared, and the abundance of *D. oblonga* decreased slightly.

**Figure 7:**
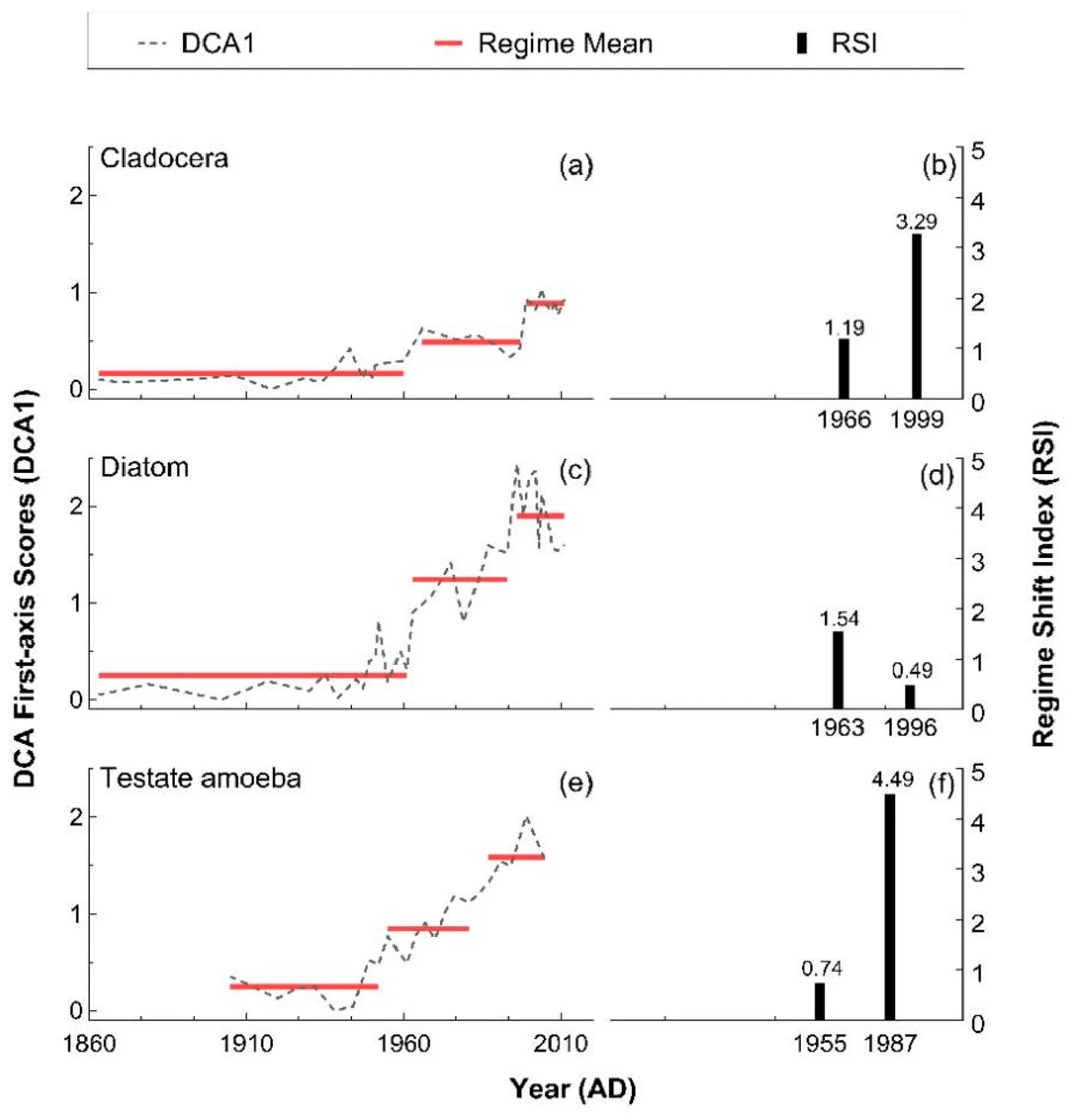
Long-term change patterns of the three biological groups: (**a**,**b**) cladocerans; (**c**,**d**) diatoms; and (**e**,**f**) testate amoebae.

STARS identified three regimes for each biological group, although the timing and magnitude of the shifts differed among the three groups (Figure 7). The DCA1 of cladocera showed a significant overall upward trend, with state mutations occurring in 1966 and 1999, corresponding RSI values of 1.195 and 3.29, respectively. The mutation intensity gradually increased, with the mean phase value rising from 0.16 to 0.89. Diatom DCA1 values generally increased across the three regimes, although individual values fluctuated. A stronger shift occurred in 1963 (RSI = 1.54), whereas the change in 1996 was much weaker (RSI = 0.49). The regime mean increased stepwise from 0.25 in the first phase to 1.89 in the third phase. The DCA1 of testate amoebae showed a sharp upward mutation trend, being the most intensely mutating group among the three, with mutation in 1955 and 1987, and RSI values reaching 0.74 and 4.49, respectively. The mean phase value rose from 0.25 to 1.59. The mutation timing of the three populations showed a temporal gradient: testate amoebae (1955) > diatoms (1963) > cladocera (1966). The mutation intensity was testate amoebae > cladocera > diatoms. Although the timing and magnitude of the shifts differed among the three biological groups, their regime means all increased stepwise across the three phases.

## 4. Discussion

### 4.1. Characteristics of the Lake Ecosystem Under Different River–Lake Connectivity Conditions

#### 4.1.1. Period of River–Lake Connectivity (1860s–1950)

Prior to 1950, the cladoceran community was primarily driven by natural factors such as summer monsoon precipitation. As a result, the flood season of the Yangtze River could last for up to 5 months, inundating adjacent floodplain systems [42]. As Lake Zhangdu was directly connected with the Yangtze River, its water level would become consistent with that of the river flooding. The P/L ratio of cladocera and the grain size, which are a reliable proxy for lake hydrodynamic intensity, both indicate that this phase was distinctly a higher-water-level period [43]. The marked increase in abundance of *Bosmina*, a small body cladoceran (0.2–1.0 mm length), during the river–lake connectivity period indicates the lake had a relatively higher trophic level [44]. Flood events delivered large quantities of nutrients into the lake system, while the intensive river–lake water exchange enhanced self-purification capacity of the water body, maintaining the water quality. Meanwhile, dominance of planktonic diatoms together with the low abundance of eutrophication-associated testate amoebae (e.g., *Difflugia corona* and *Difflugia smilion*) support self-purification capacity of the lake system during this phase [20,21].

The alpha diversity indices indicate that cladoceran diversity remained low during the period of river–lake connectivity, whereas beta diversity showed marked fluctuations, suggesting increased river–lake exchange of nutrients and biota. Except for a few species, the drastic change in hydrology during this phase may have modified habitable grounds of many cladocera in the lake [22]. Given the dominance in the abundance of *Bosmina* and *C. sphaericus*, both cladocerans could have been mediated largely by strong hydrodynamic conditions in the lake system. In addition, the higher Shannon diversity index of cladocera in 1940 corresponded to the proportion of planktonic species of cladocerans, TP and grain size fluctuations (Figure 3), a phenomenon likely caused by extreme flood events during this phase [20].

#### 4.1.2. Early Period of River–Lake Disconnection (1950–1977)

After 1950, anthropogenic factors exerted a growing influence on the cladoceran community. The P/L ratio showed that Lake Zhangdu entered a low water level, which is consistent with the historical context of the lake in this phase, as in the 1950s, reclamation and dam construction cut off the river–lake connection, leading to dramatic shrinkage of Lake Zhangdu and a significant decline in the water level [26].

A relatively weak relationship between cladocera and the environment, as shown by RDA, reflects an unusual ecosystem response to the environmental change in this phase. Only a few environmental variables, such as TP and fish yields, may have played an increased role in shaping the community structure and functioning, while other variables, including grain size and P/L ratios, decreased significantly during this period (Figure 3). A decrease in grain size and its increased negative correlation with the cladoceran community clearly corresponds to a reduced hydrodynamics condition, river–lake disconnection, intensified aquaculture, water quality deterioration and eutrophication of the lake system. Many large-scale development activities, including reclamation and aquaculture, were launched in Lake Zhangdu, introducing massive input of nutrients, salts, sediments and finer grain-size sediments and clay materials [45], causing further lake ecosystem degradation.

Intact domination of *Bosmina* and *C. sphaericus*, but gradual reversal of the relative proportion of planktonic to non-planktonic species, and a decline in planktonic diatom abundance, indicates the emergence of an unfavorable environment for planktonic reproduction (Figure 6). The river–lake disconnection is likely to have caused a sharp decline in wild fish populations, especially migratory fish species, which may have alleviated the predation pressure on *Bosmina* in Lake Zhangdu [28]. Under enriched nutrient conditions, *Bosmina* maintained its abundance. This condition is also indicated by the increased abundance of testate amoebae preferring eutrophic water [21]. *C. sphaericus* continued to increase in abundance during this phase, possibly due to its passive dispersal in nature [46], showing its life cycle to be less vulnerable to dam construction and river–lake disconnection [47]. Continuous filling of large volumes of clay and nutrients in the lake bed led to proliferate aquatic vegetation, fueling the growth of benthic cladocera such as *A. intermedia* [48]. An isolated and eutrophic lake ecosystem may have gradually transformed the lake, with a more stable cladoceran community in Lake Zhangdu being evident during this period.

#### 4.1.3. Late Period of River–Lake Disconnection (1977–1997)

During 1977–1997, the RDA results identified sediment MD, TP, and fish yield as the variables most strongly associated with changes in the cladoceran community. Fish yield increased rapidly from approximately 1.6 × 10^3^ t to 3.0 × 10^3^ t during this period, indicating intensified fishery production. This increase, however, does not directly quantify fish predation pressure.

Fish may have influenced cladocerans through both direct and indirect pathways. Planktivorous and filter-feeding fish can directly consume cladocerans and other zooplankton. Their feeding effects are often stronger on large-bodied zooplankton and may alter the size structure and composition of the zooplankton community [49]. Fish-induced reductions in zooplankton grazing may also alter phytoplankton biomass and composition, thereby changing the food resources available to cladocerans [49]. Long-term observations from Lake Donghu, another Yangtze floodplain lake, showed that increased stocking of silver carp and bighead carp strengthened top-down control and contributed to trophic-cascade effects within the lake food web [50].

Evidence from other Yangtze basin lakes also indicates that the strength of fish effects varies among lakes. Based on the surface-sediment cladoceran assemblages of 64 shallow lakes in the middle and lower Yangtze River Basin, Dong et al. [51] found that macrophyte abundance, chlorophyll a, and TP were more important in explaining cladoceran community variation, whereas fish predation showed no significant independent effect. These regional results suggest that the effects of fish on cladocerans depend on the combined influences of trophic conditions, habitat structure, and fish-community characteristics.

The Lake Zhangdu record includes total fish-yield data but lacks continuous information on fish assemblage composition. The effects of individual fish species and the relative importance of direct consumption and trophic cascades therefore remain uncertain. The concurrent increase in fish yield and changes in the cladoceran community suggest that fishery activities may have contributed to community reorganization, together with nutrient enrichment and habitat change.

The changing conditions are further indicated by the aquatic macrophyte community, such as the emergence of floating-leaved *Trapa japonica* reaching as high as 100% coverage and the decline in the submerged macrophyte community [52]. Despite this, a smaller chydorid, *A. intermedia*, adapted well to become the subdominant species in this phase, indicating they can also thrive in a reduced submerged littoral vegetation environment [22]. The increase in the relative abundance of smaller *Alona* species (*A. guttata*, *A. intermedia*, *A. rectangula*) together with benthic species *D. rostrata* clearly suggests distinct preferences of the shallow and regulated lake system [22]. A highly variable (rise-and-fall) alpha diversity in this phase suggests that eutrophication would promote species richness (Figure 3). Nutrient accumulation in the lake system may have exceeded the pollution threshold of cladocerans such as *C. sphaericus* and *Camptocercus rectirostris*, indicating the need for a species-specific conservation strategy to be adopted for lake management. The condition is also indicated by the reduced total beta diversity under high nutrient concentrations, where the cladoceran community preferring eutrophication prevailed in Lake Zhangdu (Figure 3).

#### 4.1.4. Period of Late Disconnection and Transitional Seasonal Connectivity (1997–2018)

This phase includes the final years of complete disconnection (1997–2004), followed by the seasonal connectivity restoration initiated in 2005. A substantial increase in TP and fish yield and the intensified cladocera community structure in this phase suggests widespread aquaculture activities in Lake Zhangdu. Negative correlation between DCA1 scores and fish yield further indicate ongoing management issues in the Lake Zhangdu ecosystem—for example, the need for an understanding of the food web structure and the dynamics of the lake restoration program phase [45]. The biological patterns observed in Lake Zhangdu appear to be unusually different from those of the documentary records in this phase (Figure 2). The documentary record suggests improved water quality and reduced sediment deposition after 2005 [53]. In 2005, a seasonal river–lake connection scheme, “diverting Yangtze water to replenish fish fry”, was launched, delivering an annual water replenishment of no less than 20 million cubic meters, strengthening water circulation, and possibly achieving reduced turbidity [54]. It should be noted, however, that fish-yield data are only available up to 2011 [34]. Comparable data were not available for 2013–2018, and the inferred predation pressure during this period was therefore interpreted cautiously.

The seasonal river–lake connectivity together with the complementary government measures, including the stocking of the Yangtze-origin fish fry, desilting and sand dredging, and regular harvesting of floating-leaved *Trapa*, ameliorated the conditions in Lake Zhangdu [55]. As a result, the abundant fish resources attracted large aggregations of waterbirds, including black-crowned night herons, little egrets and cattle egrets, to nest and breed in the wetland woodlands during summer, while in winter, the lake became the wintering ground for the East Asian–Australasian Flyways, indicating the importance and the complexity of the Lake Zhangdu ecosystem management and restoration. The ecological conditions in Lake Zhangdu are not only affected by external loading of large amounts of nutrients from migratory birds, but also by complex food web interactions among bird, fish and vegetation dynamics due to the seasonal river–lake connection causing occasional eutrophication [41]. It is crucial to understand that trophic levels distinctly facilitate the reproduction and survival of cladoceran communities, whereas excessive eutrophication undermines the structural stability of lake ecosystems [56,57]. The recovery of the abundance of *Bosmina* following the seasonal reconnection may indicate a biological response to renewed water exchange.

Changes observed in the uppermost samples of the 2018 core, provisionally corresponding to 2013–2018, broadly overlapped with the period following the initiation of seasonal river–lake reconnection. These patterns may indicate recent changes in the cladoceran community. However, the P/L ratio remained very low at approximately 0.8, without a clear recovery, and littoral taxa such as *Alona* continued to dominate the community. The cladoceran community had therefore not returned to its pre-1950 structure, and the habitat remained primarily littoral–benthic. Because the 2018 core lacks an independent radiometric chronology, the estimated mean sediment accumulation rate does not account for possible non-linear accumulation, top-core compaction, or disturbance during gravity coring. The exact timing of these biological changes and their relationship with the restoration measures therefore remain uncertain. The uppermost samples provide a tentative indication of recent community conditions but cannot establish the precise timing of change or a direct causal response to the restoration measures.

Our cladoceran record extends only to 2018 and therefore does not capture the ecological effects of more recent policies, particularly the Yangtze River 10-year fishing ban implemented in 2021. This policy may affect the lake food web by changing fishing pressure and fish assemblage structure, with potential consequences for zooplankton communities [58]. Future paleolimnological studies of post-2018 sediments, combined with contemporary monitoring data, are needed to evaluate the ecological changes following these conservation measures.

### 4.2. Validating Multi-Proxy Responses to River–Lake Disconnection: A Palaeoecological Synthesis

Alteration of the hydraulics and hydrology of the river system triggers ecosystem changes in the lake–sediment interface. The primary response to such a trigger comes from the benthic communities. For instance, an earlier onset of abrupt shifts in testate amoebae and submerged macrophytes in Lake Zhangdu (Figure 7) compared to all other planktonic groups, including *Bosmina*, suggests increased hydrological disturbances in the sediment–water interface [59]. Apart from the trophic position, the migration pathway and behavior, as well as the habitats, determine the temporal variability in different biological communities, including cladocerans [60]. A sudden and sharp decline in the hydrodynamic condition in Lake Zhangdu may have led to the suspended sediment settling and organic matter influx being altered due to the river–lake disconnection. These events reshaped the microhabitat structures in the sediment–water interface, indicating the complexity of the community structure dynamics of different biota. For instance, testate amoebae’s response to grain size, redox potential, and pore water nutrient concentration being one to two orders of magnitude lower than that of planktonic groups supports not only the differential role of microhabitats at the sediment–water interface but also the behavior of biota during both migration and colonization of habitats [61]. When the watershed reclamation and agricultural non-point source pollution drives sustained accumulation of nutrients in sediments, at that condition, even a minor alteration in the hydrological connectivity could disrupt material balance at the sediment–water interface, triggering a regime shift for benthic communities [62]. When water column nutrients and planktonic habitats stayed within the steady state of the ecological buffer range, benthic groups prevailed in responding to change earlier than the planktonic groups in phase one, indicating the differential behavioral responses of biota (Figure 7). For instance, diatom communities shifted abruptly a little earlier than cladocerans, showing the differential behavioral responses of planktonic ecosystems to hydrological disturbances in shallow lake systems [63].

This study suggests that diatoms, with a very short life cycle (days to weeks), are more influenced by water column nutrients, hydrodynamics, and light transparency, enabling rapid responses to environmental changes [64]. Cladocerans, on the other hand, being primary consumers, feed mainly on phytoplankton, and are influenced largely not only by the physical and chemical properties of the water column, but also by the food web structure and trophic dynamics, including fish predation pressure and interspecific competition in the lake [65]. The phenomenon of this ecological complexity among biota over time has been revealed in Lake Zhangdu under different environmental stressors, including hydrology and hydraulic changes. For instance, the ecological successions from planktonic to benthic communities observed in the 1960s were induced by the river–lake disconnection, which consequently led to a regime shift in Lake Zhangdu (Figure 6). During the succession, the first abrupt shift in testate amoebae and cladocerans clearly showed an initial response to the river–lake disconnection. Despite this response, the ecosystem remained stable due to the increased community reorganization capacity under perturbation [59]. Sustained anthropogenic disturbances such as intensive aquaculture development, continuous nutrient accumulation, and aggravated lake paludification in Lake Zhangdu led to depleted buffering capacity, followed by the emergence of a second abrupt shift in 1987. The shift this time indicates that the biota had crossed a threshold, and the failure of the system to reorganize the community led not only to the total extirpation of keystone species (e.g., *Difflugia penardi*) but also to the emergence of more pollution-tolerant species in Lake Zhangdu (Figure 7).

The low RSI value of 0.49 indicates that the 1996 change in the diatom community was weak. We interpret this change as a minor community adjustment within the longer-term pattern. Its ecological significance remains uncertain, and it is not treated as a major regime transition or tipping point in this study. Characterizing microhabitats and their niche attributes will, however, become essential to understanding the abrupt shifts in testate amoebae in Lake Zhangdu in response to anthropogenic stress. Their microhabitat, composed of sediments, is exposed to a range of anthropogenic disturbances, including hydrological disconnection, nutrient burial, organic matter degradation, and heavy metal accumulation. After decades of continuous impacts, the degree of microhabitat alteration in sediments may have become far greater than in the open-water habitat [61]. It is crucial to know that, due to having extremely narrow niche breadths and highly specific preferences for habitat and physicochemical parameters in sedimentary environments, the testate amoeba community can show variable tolerance capacities, often tending to trigger the largest ecological regime shift [66]. Differential responses of the three biological groups to hydrodynamic conditions, including the river–lake disconnection in Lake Zhangdu, provide an understanding of the significance of microhabitats, migratory pathways and species-specific biological behavior in responding to environmental change during ecological succession. Key microhabitats such as the sediment–water interface for benthic communities (testate amoebae and submerged vegetation) and the surface water column for planktonic communities (*Bosmina* and diatoms), as well as the behavioral responses to hydraulics and hydrodynamic conditions, play a significant role in the ecological reorganization of community structuring. However, sustained anthropogenic disturbances in the lake system would lead to threshold crossing followed by an abrupt shift and a possible irreversible ecological regime with limited ecosystem services. Using the divergence of paleoecological indicators from different taxa is crucial while developing more effective shallow lake management strategies [67].

## 5. Conclusions

Cladoceran subfossils retrieved from Lake Zhangdu provide an important record of its environmental history over the past 150 years. Their response to the transitioning lake ecosystem at the time of critical anthropogenic disturbance such as disruption in the river–lake connectivity is crucially useful in the context of shallow lake management in China. The diversity assemblages and P/L ratios of cladocera under hydraulic and hydrological condition changes in the river system would directly affect microhabitat and trophic dynamics, triggering a regime shift. Hence, the river–lake disconnection in Lake Zhangdu is a fundamentally important phenomenon from the management perspective. The use of multiproxy approaches, including biological community responses, is essential to validate the anthropogenic impact on the lake ecosystem. However, marked divergences in timing, magnitude and underlying driving forces of cladocerans, diatoms and testate amoebae observed in Lake Zhangdu suggest the importance of species-specific niche traits, micro-habitats and behavioral responses, including tolerance and thresholds to environmental stressors, should be considered while developing the management strategies. This study shows that the use of multiple proxy indicators can provide a strong evidence base for formulating restoration and management measures for degraded lake ecosystems and help overcome the limitations of relying on a single indicator.

## Data Availability

The original contributions presented in this study are included in the article. Further inquiries can be directed to the corresponding authors.

## Abbreviations

The following abbreviations are used in this manuscript:

WWF	World Wide Fund for Nature
TLI	Total Nutrient Load Index
LOI	Loss on Ignition
βsor	Sørensen dissimilarity index
TOC	Total Organic Carbon
TP	Total Phosphorus
χfd	frequency-dependent magnetic susceptibility
DCA	Detrended Correspondence Analysis
RDA	Redundancy Analysis
STARS	Sequential *t*-test Analysis of Regime Shifts
RSI	Regime Shift Index
P/L	Planktonic-to-littoral cladoceran ratio
MD	Median Diameter
